# Theoretical investigation of a plasmonic substrate with multi-resonance for surface enhanced hyper-Raman scattering

**DOI:** 10.1038/s41598-018-30331-x

**Published:** 2018-08-08

**Authors:** Shuangmei Zhu, Chunzhen Fan, Pei Ding, Erjun Liang, Hongwei Hou, Yuanda Wu

**Affiliations:** 1 The College of Chemistry and Molecular Engineering, Zhengzhou University, Zhengzhou, 450001 China; 2 College of Science, Henan University of Engineering, Zhengzhou, 451191 China; 3 Henan Shijia Photons Technology Co., Ltd, Hebi, 458030 China; 4 School of Physical Science and Engineering and Key Laboratory of Materials Physics of Ministry of Education of China, Zhengzhou University, Zhengzhou, 450052 China; 5 School of Science, Zhengzhou University of Aeronautics, Zhengzhou, 450046 China

## Abstract

Because of the unique selection rule, hyper-Raman scattering (HRS) can provide spectral information that linear Raman and infrared spectroscopy cannot obtain. However, the weak signal is the key bottleneck that restricts the application of HRS technique in study of the molecular structure, surface or interface behavior. Here, we theoretically design and investigate a kind of plasmonic substrate consisting of Ag nanorices for enhancing the HRS signal based on the electromagnetic enhancement mechanism. The Ag nanorice can excite multiple resonances at optical and near-infrared frequencies. By properly designing the structure parameters of Ag nanorice, multi- plasmon resonances with large electromagnetic field enhancements can be excited, when the “hot spots” locate on the same spatial positions and the resonance wavelengths match with the pump and the second-order Stokes beams, respectively. Assisted by the field enhancements resulting from the first- and second-longitudinal plasmon resonance of Ag nanorice, the enhancement factor of surface enhanced hyper-Raman scattering can reach as high as 5.08 × 10^9^, meaning 9 orders of magnitude enhancement over the conventional HRS without the plasmonic substrate.

## Introduction

Unlike normal Raman scattering (RS) that resulting from the scattering of a single photon, hyper-Raman scattering (HRS) is an inelastic sum-frequency scattering from two photons. The two photons with frequency of *ν* are inelastically scattered from a ground state to a virtual state with energy equal to *2ν* − *ν*_*vib*_ or *2ν* + *ν*_*vib*_, corresponding to Stokes and anti-Stokes scattering, respectively, where *ν*_*vib*_ is vibrational frequency^1,2^. Depending on the symmetry of molecular, HRS may probe Raman and infrared active modes as well as the so-called silent modes that are seen neither in Raman nor in infrared spectrum. The silent modes can offer complementary vibrational information that can’t be revealed by both RS and infrared absorption^3^. Because of this capability to provide better insight into the structure and interaction of molecules, HRS is considered as a spectroscopic technique with more sensitive than RS with respect to surface environmental changes^4^.

However, due to the two-photon process and the little cross-sections of about 10^6^ times weaker than RS, the experimental detection for HRS is very difficult^5^. For a long time in the past, HRS has not been considered as an applied spectroscopic technique until people discovered surface enhancement Raman scattering (SERS) phenomenon. Inspired by SERS, surface enhanced hyper-Raman scattering (SEHRS) effect has been realized by enhancing the weak hyper-Raman scattering through the strong local electric field that is generated by exciting localized surface plasmon resonances (LSPRs) in metal nanostructures (Au, Ag, Cu, etc.)^6–10^. With the development of experimental technology and theoretical research, SEHRS spectrum has attracted great attention and been used in many fields^11–14^, such as single molecule detection^11^, cell PH sensors^15^, spectral imaging like other nonlinear optical imaging^16,17^.

**Figure 1:**
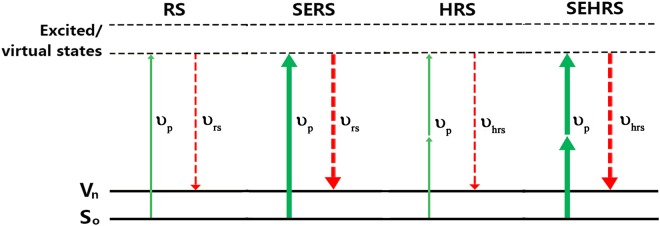
Energy-level diagrams for Raman scattering, surface-enhanced Raman scattering, hyper-Raman scattering, and surface-enhanced hyper-Raman scattering.

The electromagnetic field enhancement factor (EF) in SEHRS can be estimated as follows^18^:

1 $$E{F}_{SEHRS}^{EM}={|E(\nu )/{E}_{0}(\nu )|}^{4}{|E({\nu }_{s})/{E}_{0}({\nu }_{s})|}^{2}={|g(\nu )|}^{4}{|g({\nu }_{s})|}^{2}$$

where *ν* and ${\nu }_{s}({\nu }_{s}=2\nu -{\nu }_{vib})$ are the frequency of incident light and scattering light, respectively. |*g*| is the local electric field enhancement at the location of probe molecule. Different from SERS, SEHRS has higher-order dependence on the incident light intensity, thus indicates higher enhancement factor than SERS. Figure 1 shows the enhancement mechanism of SEHRS and the comparison with SERS. From equation (1), an optimum plasmonic substrate for SEHRS applications require that (i) the significant electric-field enhancements occur simultaneously at two different spectral regions around *ν* and *ν*_*s*_; and (ii) the electric hotspots generating at the two spectral regions should overlap spatially, i.e. in the same spatial locations. Both are indispensable. However, for a regular plasmonic substrate, electromagnetic enhancement resulting from |*g*(*ν*)|^4^ and |*g*(*ν*_*s*_)| ^2^ are not easy to be obtained at the same time, because different surface plasmon resonances usually have different “hot spot” locations. For SERS, a single plasmon resonance is broad enough to enhance both the incident and Raman scattering fields. But for SEHRS, the plasmonic substrates should support double-resonances with the same “hot spot”. Therefore, in contrary to its linear counterpart with rapid developments, SEHRS has only been sparsely studied despite the great potential for detecting low energy molecular vibrations and vibrational molecular modes inactive in both RS and infrared absorption^19–22^. In order to promote the application and development of SEHRS technology in different fields, it is particularly important to develop SEHRS substrates which can enhance the incident light and the scattered light in the same spatial locations. In fact, for surface enhanced nonlinear optical processes, the plasmonic substrate with multiple resonances at the same spatial locations is indispensable^23,24^.

Precise control of the size and morphology of metallic nanostructures is critical for tuning the LSPR energy and intensity as well as improving the efficiency of light manipulation^25^. Silver or gold nanorice structures support both transverse and longitudinal resonances, with the latter tunable from the visible to the near-infrared spectral range by variation of the aspect ratio (length/diameter)^26,27^. With increasing aspect ratio of nanorice, higher-order multipolar LSPR modes can be excited^28,29^, which facilitates their applications in ultrasensitive sensing^28^, surface-enhanced Raman spectroscopy^29^, and catalysis^30^. These current studies of silver or gold nanorices won’t pay attention to multi-resonance phenomenon with the spatial overlap of the electric hotspots for surface enhancement hyper-Raman scattering.

In this article, we numerically studied a kind of plasmonic substrate for SEHRS application. The plasmonic substrate consists of Ag nanorices that supporting multi-resonances. It is found that two of the excited plasmon modes not only have large field enhancements at the same “hot spots” but also spectrally match with the excited light and second-order Stokes scattering. Therefore, the Ag nanorice is expected to be used for enhancing the SEHRS process. By investigating the far-field scattering spectra and the near-field hot-spot distribution of the nanorice with different length or diameter, we discussed the influence of geometric parameters on the signal enhancement of HRS. The theoretical value of maximum EF for SEHRS can reach 5.08 × 10^9^. Our study is expected to provide theoretical supports for fabricating superior SEHRS substrate for single molecule detection and unknown molecular recognition.

## Multipolar Plasmonic-Resonant Structure

**Figure 2:**
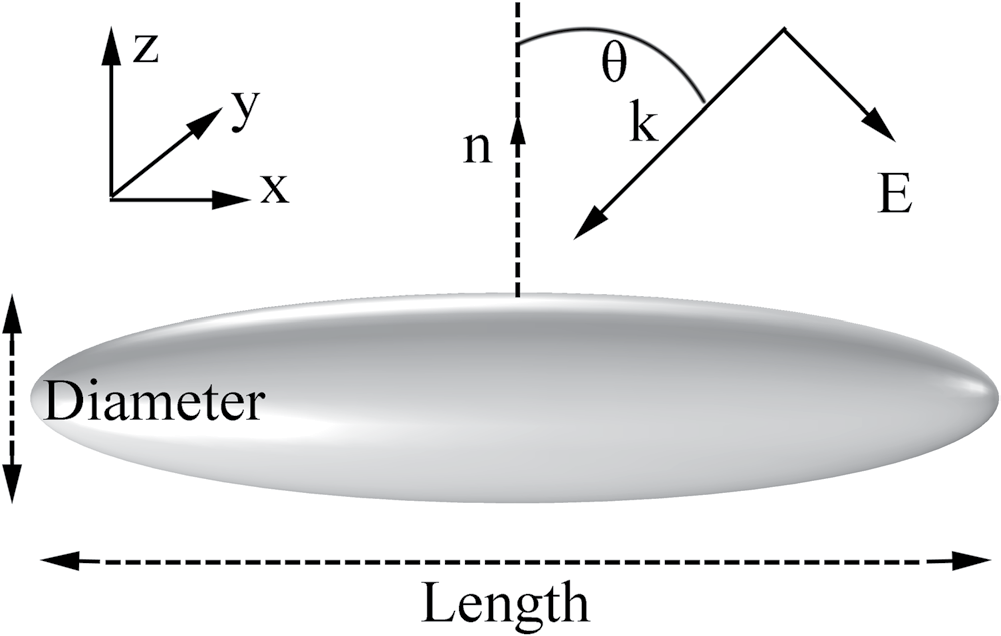
Schematic of the Ag nanorice structure. The Ag nanorice locates in the x-y plane, with its normal direction n along the z axis. Linearly polarized light illuminates this structure at normal incidence (θ = 0°) or oblique incidence (θ > 0°).

The schematic of nanorice is shown in Fig. 2, where both the geometry parameters and the incident light polarization configuration are labeled. A plane wave is incident with an angle of θ to the normal direction^31^. All calculations about the nanorice, including the scattering spectra and the electric field distributions, were carried out by COMSOL Multiphysics 5.2, a commercial three-dimensional numerical simulation software based on finite-element method (FEM). Perfect matched layers (PML) were employed for surrounding boundaries to avoid spurious reflections. For simplification, the isolated Ag nanorice was assumed to be in air with the refractive index *n* = 1. Introducing dielectric substrates in practice for probe molecules does not modify the optical properties of the nanorice, but only shifts the resonance to longer wavelengths together with a slight increase in linewidth^32,33^. The permittivity of Ag nanorice was taken from the experimental data given by Johnson and Christy^34^. Scattering cross sections were computed according to scattering formulation: ${\sigma }_{sc}=\int \int \mathop{n}\limits^{\rightharpoonup }\cdot {\mathop{S}\limits^{\rightharpoonup }}_{sc}dS$^35^. During the calculation, a good convergence was obtained by utilizing adaptive meshing technique to handle the structure with large aspect ratios. We calculated the scattering spectra and electric field distributions of the nanorice with the total length (L) and maximum diameter (d), as shown in Fig. 2.

## Results and Discussions

### Spectral tunability of the multipolar resonances

**Figure 3:**
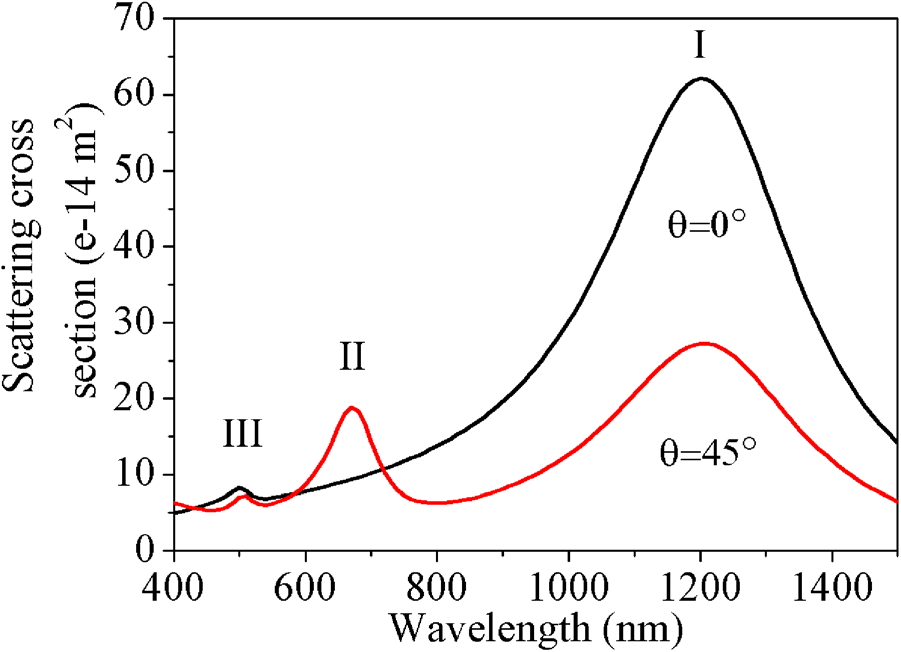
Scattering spectra of an individual Ag nanorice under normal (θ = 0°, black) or oblique (θ = 45°, red) incidence.

The localized field enhancement caused by plasmon resonance contributes greatly to the light-matter interactions at the nanoscale, especially when the probed molecules are located at the “hot spots”. The Ag nanorice with length of L = 460 nm and diameter of d = 100 nm was used to excited multiple LSPRs. The simulated scattering spectra of the nanorice under normal and oblique incidence are shown in Fig. 3. The scattering spectra indicate that as the incident angle is *θ* = 0°, the nanorice has two distinct resonances at the wavelength of 1210 nm and 510 nm, corresponding respectively to the first- (I) and the third-longitudinal resonance (III) mode of the nanorice. While for *θ* = 45°, there are three resonances, appearing at the wavelength of 1210 nm, 670 nm and 510 nm and corresponding to the first three multi-resonance modes, respectively. According to the electromagnetic enhancement mechanism and Equation (1), to achieve a significant enhancement of HRS signal, the spectral positions of the multi-resonance modes and the exciting and scattering lights should match. We noted that the wavelengths (1210 nm and 670 nm) of the first- and second-longitudinal resonance just match with that of the exciting light and the second-order Stokes light of SEHRS^36^.

**Figure 4:**
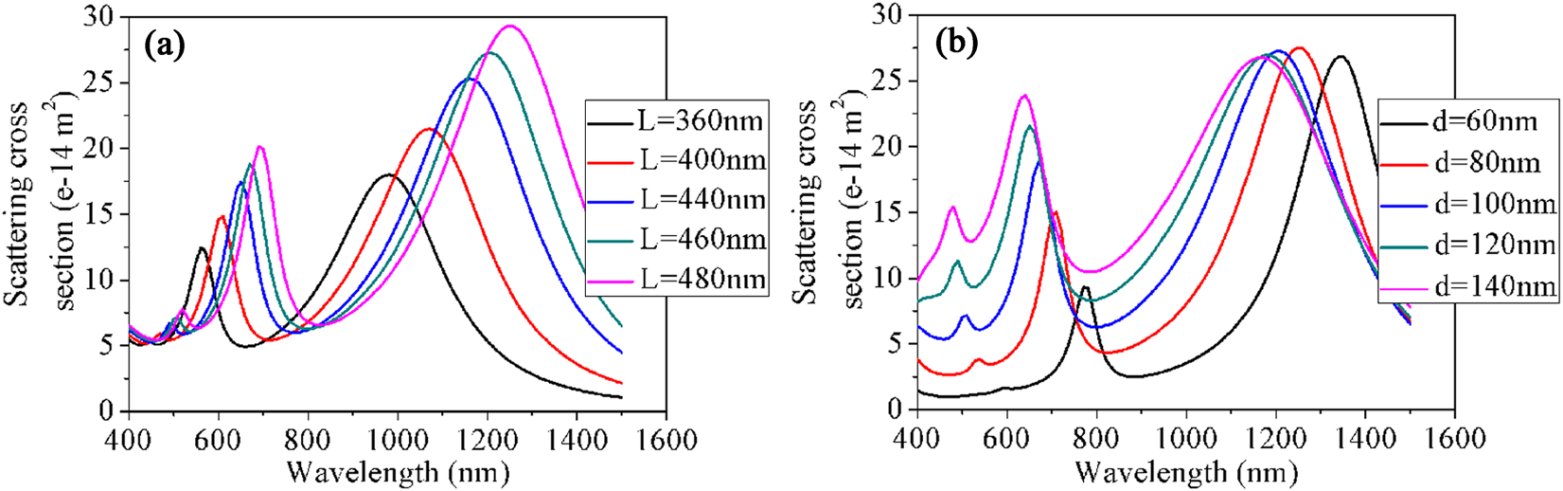
Scattering spectra of the nanorice with different geometrical parameters. (**a**) Varying the length (L) as the diameter is fixed (d = 100 nm). (**b**) Varying the diameter (d) as the length is fixed (L = 460 nm).

Changing the value of length and diameter of the nanorice can shift the spectral position of multi-resonance^27^. Figure 4 shows the scattering spectra of the nanorice with different geometrical sizes under oblique incidence (θ = 45°). In Fig. 4(a), when the geometrical parameter L was increased from 360 to 480 nm, the first two scattering peaks all shift toward long wavelengths, but the redshift is more obvious for the dipole resonance mode. Conversely, the two resonances shift toward short wavelengths when the geometrical parameter d increases from 60 to 140 nm, as shown in Fig. 4(b). In addition, the second- and third-longitudinal resonance peaks at the wavelength of 670 nm and 510 nm become more obvious with the increase of d. That is the high-order mode of the surface plasmon resonances become stronger as the diameter of nanorice increase. However, the first-longitudinal resonance mode have little changes on the peak value, but show an obvious increase in the linewidth, which means that the first resonances will have smaller quality factor and thus weaker local electric field with the increase of d^37^.

### SEHRS enhancement

**Figure 5:**
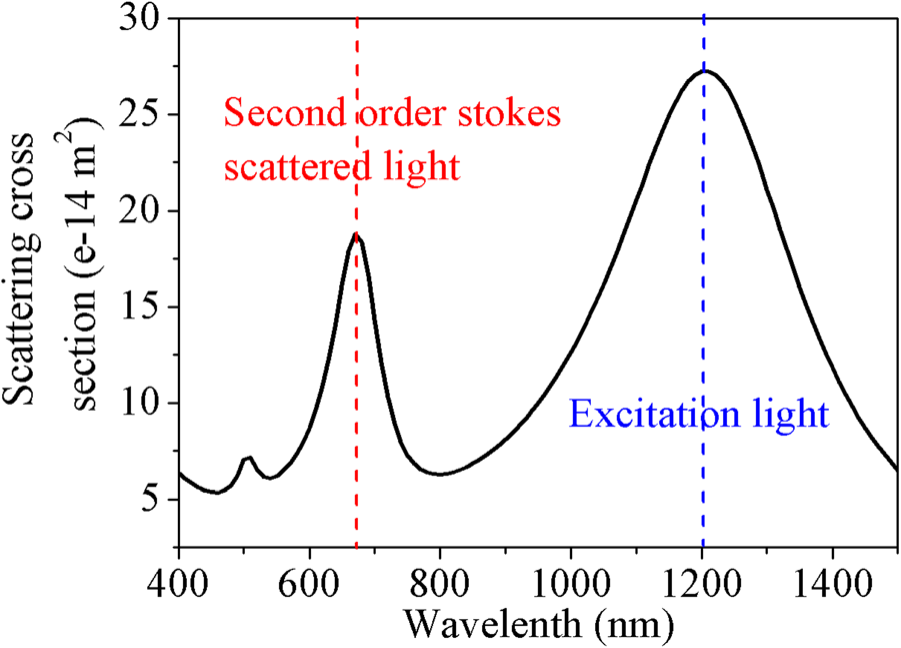
Scattering spectrum of a nanorice with optimized geometrical parameters (*L* = 360 nm, *d* = 100 nm) for enhancing the 4MBA 1590 cm^−1^ mode with a 1210 nm excitation light. Here, the first- and the second-longitudinal resonance are just matched to the wavelengths of excitation (blue, 1210 nm) and second-order Stokes (red, 670 nm) light, respectively.

To evaluate the SEHRS enhancement caused by the multipolar plasmonic resonances in Ag nanorice, we next calculate the distribution of electic-field and the value of EF for a Raman mode of 4-mercaptobenzoic acid (4MBA) molecules at 1590 cm^−1^. The formula between the hyper-Raman frequency shift and the wavelength is as follows:

2 $${\lambda }_{{\rm{s}}}=\frac{{10}^{7}}{\frac{{10}^{7}}{{\lambda }_{{\rm{ex}}}\,/\,2}-{\rm{\Delta }}\upsilon }$$

where, *λ*_ex_ and *λ*_*s*_ are the wavelength of incident light and scattering light, respectively, and their units are nanometer. Δν is hyper-Raman frequency shift, and its unit is inverse centimeter. According to equation (2), when the exciting light is assumed at the wavelength of 1210 nm, the corresponding second-order Stokes wavelength should be around 670 nm. By optimizing the geometrical parameters of Ag nanorice, the wavelengths of dipole resonance and second-order resonance can be adjusted to match with that of the exciting light and the second-order Stokes scattering light (see Fig. 5). It would be advantageous for the enhancement of SEHRS, because at round the resonance wavelengths the local electric fields have maximum enhancement.

**Figure 6:**
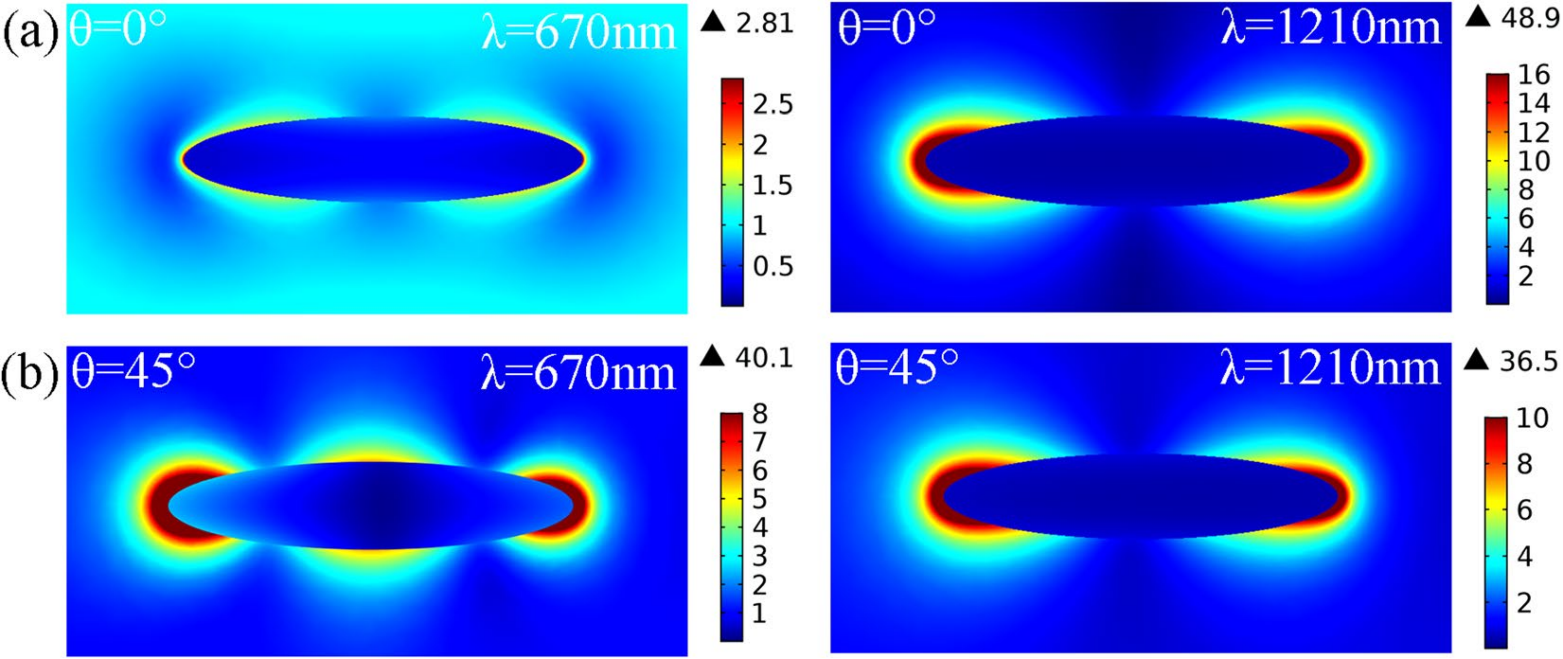
Distributions of electric field amplitude (|*E*/*E*_0_|) of the Ag nanorice at the wavelengths of excitation light (1210 nm) and second-order Stokes (670 nm) scattered light under (**a**) normal, or (**b**) 45°-oblique incidence.

Coherent oscillation of two distinct resonances in the same spatial location is highly desired for SEHRS substrates^38^. However, this is generally impossible for a simple plasmonic structure. In Fig. 6(a), when the incident angle is θ = 0°, a significant enhancement of electric field with the “hot spots” in the ends of the long axis of the nanorice were observed for the first-order resonance mode at the wavelength of 1210 nm. But there is a weak enhancement for the second-longitudinal resonance at the wavelength of 670 nm, which is disadvantage for SEHRS. However, it is different for the case of oblique incidence, because the oblique incidence can break the symmetry of the configuration and have some multipolar resonance mode be excited^39^. For θ = 45°, significant “hot spots” at the ends of nanorice can also be seen clearly for the second-order mode (670 nm), which overlaps with those of the first-order resonance (1210 nm), as shown in Fig. 6(b). Resulting from the electric field enhancement occurring at the same “hot spots” but two different wavelengths, the signal intensity of SEHRS will be enhanced as discussed above.

**Figure 7:**
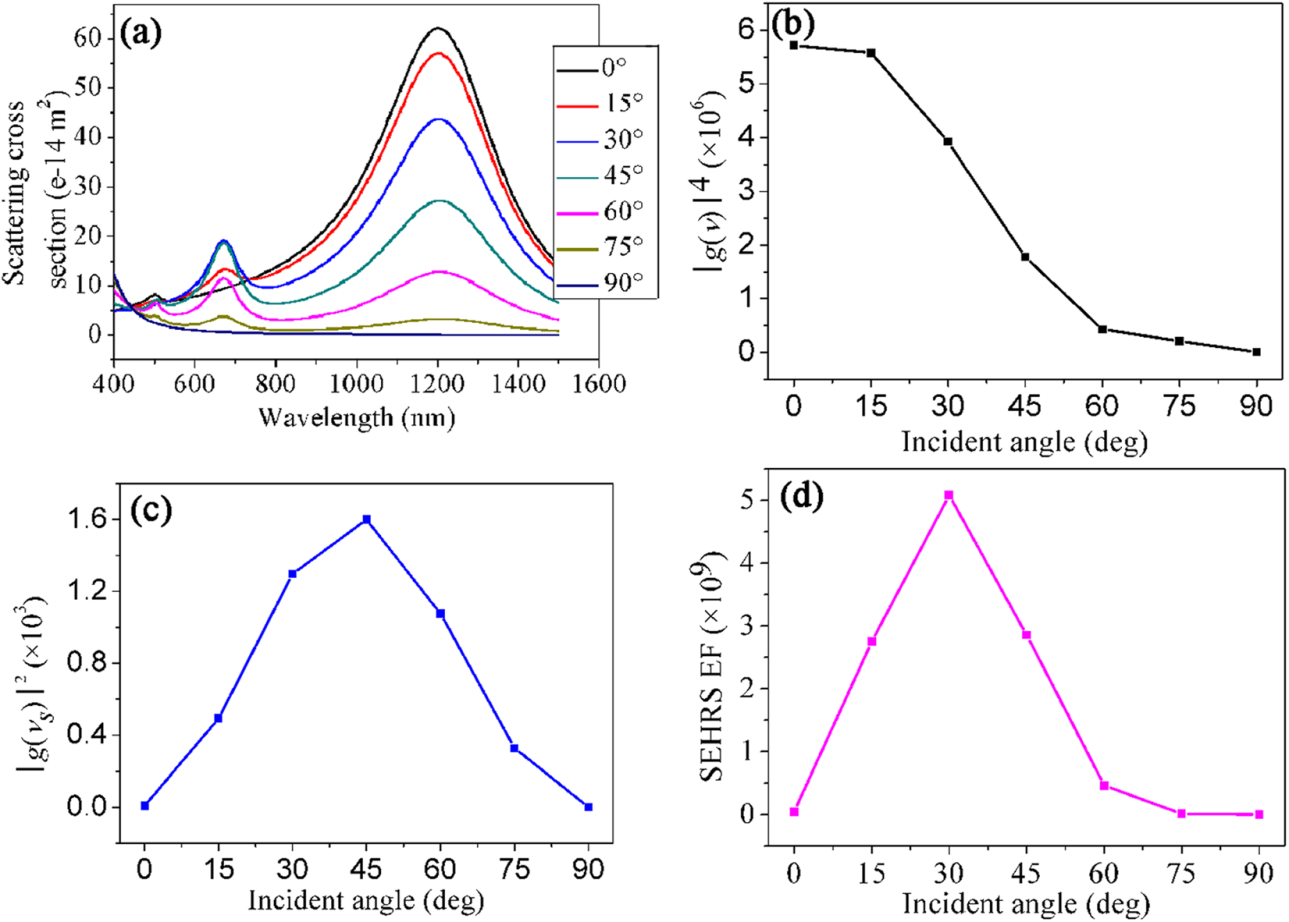
(**a**) Scattering spectra of the nanorice (*L* = 460 nm, *d* = 100 nm) as the incident angle increase from 0° to 90° with an increment of 15°, (**b–d**) excitation enhancement (|*g*(*ν*)|^4^), scattering enhancement (|*g*(*ν*_*s*_)|^2^) and the maximum value of EF for SEHRS of the nanorice under various incident angles.

Next, we investigate the dependence of the scattering spectra and the EF of SEHRS on the incident angle of a plane wave for an Ag nanorice substrate. Figure 7(a) displays the scattering spectra of the nanorice structure as the excitation angles changing from 0° to 90°. It can be seen clearly that as the incident angle increases, the relative intensity of the first-longitudinal resonance (~1210 nm) gets weak while the second-longitudinal resonance (~670 nm) is firstly strengthened and then weakened. At 0° and 90°, the second-longitudinal resonances are completely suppressed. According to the scattering spectrum, the resonance frequency, the linewidth and the quality factor of the surface plasmon resonance can be determined, which can directly indicate the far-field performance of the resonance mode and simultaneously give some insights on near-field effect^37^. However, an accurate estimate of EF of SEHRS should base on the enhancement of the localized electric field. According to equation (1), the value of EF, i.e. SEHRS enhancement factor can be obtained by calculating the electric-field enhancements of the first- and the second-longitudinal resonance at 1 nm from the end of the nanorice. Figure 7(b,c) shows the calculated results of excitation enhancement (|*g*(*ν*)|^4^) and scattering enhancement (|*g*(*ν*_*s*_)|^2^), which are contributed by the resonances at the wavelength of 1210 nm and 670 nm, respectively. The calculated values of the maximum EF under different incident angles are shown in Fig. 7(d). The value of EF, firstly increases, reaching the maximum at about 30°, and then diminishes significantly with the incident angle. The small value of EF at θ = 0° or 90° is mainly because the second-longitudinal resonance can’t be excited effectively, as indicated in Fig. 6. Through the above analysis, SEHRS EF reaches the maximum of 5.08 × 10^9^ at the incident angle of 30°. This indicates that the SEHRS signal is ~9 orders of magnitude larger than the standard HRS, reaching the sensitivity of single-molecule detection^40,41^. By contrast, for normal incidence θ = 0°, when the scattering enhancement is small and the field optimization is not achieved, the calculated EF of SEHRS is only 4.51 × 10^7^ (|*g*(*ν*)|^4^|*g*(*ν*_*s*_)|^2^ = 48.9^4^ × 2.87^2^ = 4.51 × 10^7^). Therefore, the EF of SEHRS under the case of θ = 30° (5.08 × 10^9^) is ~113 times larger than that of θ = 0° (4.51 × 10^7^), which demonstrates that the plasmonic substrate with significant field enhancement at the excitation and simultaneously the scattering wavelength is very important for SEHRS applications.

## Conclusions

We have theoretically investigated a SEHRS substrate consisting of Ag nanorice with multi-resonance. By exciting the first- and second-longitudinal plasmon resonance, we demonstrate this plasmonic structure can generate electric field “hot spots” at the same spatial locations when different resonance modes are excited. The resonance frequencies of the two modes match exactly with the excited light and the second-order Stokes scattering. The electric field “hot spots” of the nanorice excited at the specific spectral position can be tuned actively by changing the excitation orientation of the plane wave. Due to the improvement of both the excitation and the emission enhancement in SEHRS process, the theoretically predicted EF for SEHRS can reach 5.08 × 10^9^ at the incident angle of 30°, getting the sensitivity for single-molecules detection. The plasmonic substrate with multi-resonance developed here also holds promise for other applications, such as other nonlinear spectroscopy, stimulated Raman scattering and multiphoton imaging.
